# Characterization of Magnetoresistive Shunts and Its Sensitivity Temperature Compensation

**DOI:** 10.3390/s24103047

**Published:** 2024-05-11

**Authors:** Diego Ramírez-Muñoz, Rafael García-Gil, Susana Cardoso, Paulo Freitas

**Affiliations:** 1 Department of Electronic Engineering, University of Valencia, Avda. de la Universitat, s/n, 46100 Burjassot, Spain; ramirez@uv.es; 2 INESC Microsistemas e Nanotecnologias (INESC-MN) and Instituto Superior Tecnico, Universidade de Lisboa, R. Alves Redol 9, 1000-029 Lisbon, Portugal; scardoso@inesc-mn.pt (S.C.); pfreitas@inesc-mn.pt (P.F.)

**Keywords:** magnetoresistive shunt, current sensor, magnetoresistance, shunt, temperature compensation

## Abstract

The main purpose of the paper is to show how a magnetoresistive (MR) element can work as a current sensor instead of using a Wheatstone bridge composed by four MR elements, defining the concept of a magnetoresistive shunt (MR-shunt). This concept is reached by considering that once the MR element is biased at a constant current, the voltage drop between its terminals offers information, by the MR effect, of the current to be measured, as happens in a conventional shunt resistor. However, an MR-shunt has the advantage of being a non-dissipative shunt since the current of interest does not circulate through the material, preventing its self-heating. Moreover, it provides galvanic isolation. First, we propose an electronic circuitry enabling the utilization of the available MR sensors integrated into a Wheatstone bridge as sensing elements (MR-shunt). This circuitry allows independent characterization of each of the four elements of the bridge. An independently implemented MR element is also analyzed. Secondly, we propose an electronic conditioning circuit for the MR-shunt, which allows both the bridge-integrated element and the single element to function as current sensors in a similar way to the sensing bridge. Third, the thermal variation in the sensitivity of the MR-shunt, and its temperature coefficient, are obtained. An electronic interface is proposed and analyzed for thermal drift compensation of the MR-shunt current sensitivity. With this hardware compensation, temperature coefficients are experimentally reduced from 0.348%/°C without compensation to −0.008%/°C with compensation for an element integrated in a sensor bridge and from 0.474%/°C to −0.0007%/°C for the single element.

## 1. Introduction

Current sensing is required in many power electronic applications for control, power management, overcurrent protection, or monitoring tasks [1,2]. Techniques used in measuring electric current include, mainly: shunt resistors, current transformers, Rogowski coils, Hall effect sensors, and magnetoresistive (MR) sensors [3]. Shunt resistor-based techniques use an external resistor to measure current, which is dissipative and does not provide galvanic isolation [1,4]. Current transformers use a magnetic core with a large number of secondary turns, the primary being winding the conductor through which the current to be measured circulates. This solution inherently provides galvanic isolation, but it requires large cores for low-frequency currents and precludes the measurement of DC currents [4,5]. The Rogowski probe is a flexible coil, without a magnetic core, used to measure AC currents in high-power systems, providing accurate readings without introducing a significant impedance. These sensors have galvanic isolation and a wide frequency response; however, they do not allow the measurement of DC currents [3,4,6]. Hall effect sensors used for current measurement consist in semiconductor materials that generate a voltage proportional to the current passing through a conductor when exposed to a magnetic field [7]. Hall sensors offer non-contact current sensing, galvanic isolation, wide dynamic range, compact size, low power consumption, and fast response time, but they have limitations in accurately measuring high-frequency currents, have low sensitivity, and their sensitivity is affected by changes in temperature [8]. MR sensors, used in this work, vary their resistance depending on the applied magnetic field and, therefore, depending on the current to be measured. Although Hall effect sensors are the most used in power electronics applications, MR sensors offer advantages in terms of sensitivity and compact size [2].

Three different technologies of magnetoresistances have been applied in current sensing: anisotropic magnetoresistance (AMR) [9], giant magnetoresistance (GMR) [10], and tunnel magnetoresistance (TMR) [2,11]. The TMRs are better in terms of temperature stability, power consumption, and size [2].

MR sensors are generally integrated into a Wheatstone bridge (WB) structure made up of four out-of-phase sensing elements [12,13,14,15]. Traditionally, this configuration has been used in order to reduce the offset term and thus be able to amplify the obtained signal with an instrumentation amplifier with enough gain, while achieving linearity, noise reduction, and reduced temperature coefficient (TC) of sensitivity [15]. However, ensuring symmetrical resistance variation between the branches of the bridge in the implementation adds complexity to the manufacturing process, increasing production costs [13]. Furthermore, MR sensors have sensitivity thermal dependence, which requires some type of thermal compensation (hardware or software). Thermal compensation strategies for MR sensors integrated in a WB structure can be found in [16,17,18,19].

In the literature, there have been efforts to reduce the number of active elements in the magnetoresistive bridge to cut down production costs [13,15,20,21]. However, the combined integration of passive and active elements introduces discrepancies and inhomogeneities that reduce the Wheatstone bridge performance [13].

Using a single active element would be a viable solution for reducing production costs and layout complexities while avoiding the discrepancies that appear when active and passive elements are combined in a WB. In [22], a single active element is used instead of a WB for piezoresistive sensors applied in cardiac catheters with the purpose of reducing sensor area, demonstrating a good performance in terms of sensitivity. A single element for piezoresistive sensors is also proposed in [23], where a temperature analysis is also included. In this work, the voltage–pressure curves, measured at different temperature conditions, exhibit discernible voltage gaps between them. 

For MR sensors, papers [12,15,20,21,24] explore the potential use of a single active element, but there is a lack of experimental studies regarding their characterization and thermal dependence. Additionally, no efforts have been made to propose electronic conditioning or temperature compensation strategies.

This paper explores the feasibility of using an MR element as a current sensor. When the MR element is biased at a constant current, the voltage drop across its terminals provides information, by the MR effect, of the current to be measured, in a similar way to a conventional shunt resistor. However, the MR-shunt offers the advantage of being a non-dissipative shunt, as the current of interest does not circulate through the material, avoiding self-heating. Moreover, it provides galvanic isolation and enables the measurement of both AC and DC currents. The operational similarities of the proposal with the shunt resistor allow us to introduce the concept of a magnetoresistive shunt (MR-shunt). Furthermore, in this paper, the authors carry out a thermal characterization of the sensitivity of the elements and propose an electronic interface which includes the hardware circuitry for thermal compensation of their sensitivity. An appreciable reduction inf the temperature coefficient for different elements is obtained, showing the validity of the MR-shunt as a current sensor.

This paper is organized as follows. Section 2 presents the two MR elements analyzed, one embedded into a Wheatstone bridge and another independently implemented. In this section, an electronic circuitry enabling the utilization of the available MR sensors embedded into a Wheatstone bridge as sensing independent elements is also introduced. In Section 2.1, the proposed conditioning circuit enabling the MR element to function as a current sensor is analyzed. The proposed thermal compensation method is explained in Section 2.2. Section 3 outlines the experimental results, including the validation of the electronic interface, measurements of the sensitivity temperature coefficient without compensation, and the outcomes when the proposed compensation is implemented. In Section 4, a brief discussion takes place, while in Section 5, conclusions are drawn.

## 2. MR-Shunt Definition and Thermal Characterization

In this paper, tunnel magnetoresistance (TMR) effect-based sensing elements, both arranged in a Wheatstone bridge configuration and single element, were used. Sensors micro-fabrication was carried out at the INESC-MN facilities in Lisbon. In the case of the magnetoresistive bridge, named TMR46, each element was made up of the following layers (thicknesses in nm): Si/100 SiO_2_/5 Ta/15 Ru/5 Ta/15 Ru/5 Ta/5 Ru/20 IrMn/2 CoFe_30_/0.85 Ru/2.6 CoFe_40_B_20_/1.8 MgO/2 CoFe_40_B_20_/0.21 Ta/4 NiFe/0.20 Ru/6 IrMn/2 Ru/5 Ta/10 Ru. The stand-alone MR element, named LCEL1, has the structure: (5 Ta/10 Ru)x3/5 Ta/5 Ru/20 MnPt/2.2 Co_80_Fe_20_/0.7 Ru/1.8 CoFeB/1.8 MgO/3 CoFeB/0.2 Ru/6 NiFe/6 MnIr/5 NiFe/5 Ru/5 Ta/10 Ru.

**Figure 1:**
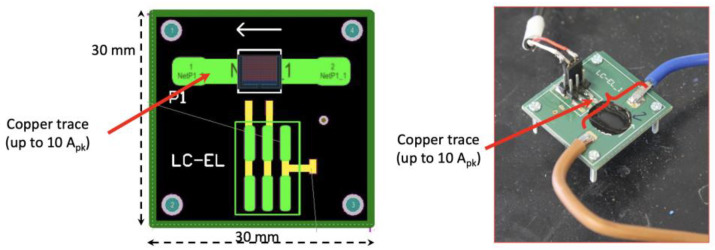
Practical implementation of an MR-shunt.

Figure 1 shows the layout of the tracks and their implementation for the single element (LCEL1). In the case of the magnetoresistive bridge, the current passes through a U-shaped copper bar located at the bottom of the PCB, while in the case of the single element, a properly designed single track on the PCB was used.

**Figure 2:**
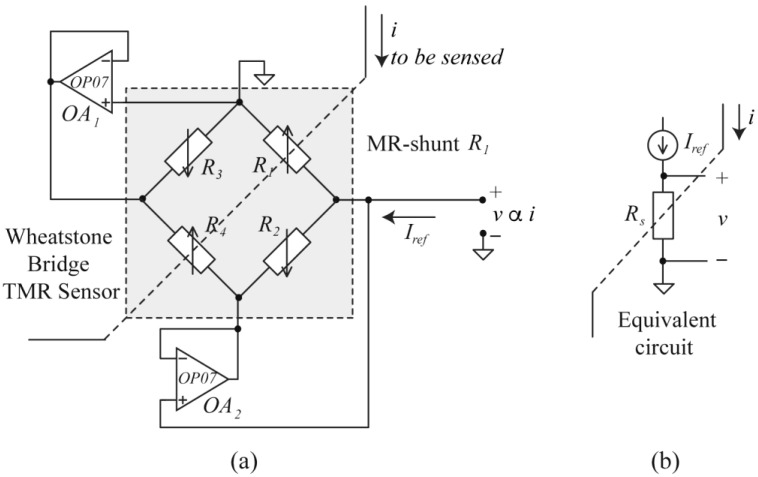
(**a**) Electronic circuitry to reduce an MR Wheatstone bridge sensor to an MR-shunt and (**b**) its equivalent circuit.

In order to be able to use the TMR sensors configured in a Wheatstone bridge as an MR-shunt, a circuit was devised that allows the rest of the elements to be cancelled. The circuit is shown in Figure 2a, where sensor elements *R*_2_, *R*_3_, and *R*_4_ are eliminated (in this particular case), leaving only element *R_1_* as the active element. By action of operational amplifiers *OA*_1_ and *OA*_2_, current through *R*_2_ and *R*_3_ elements is zero and, therefore, also zero through *R*_4_. As a consequence, only element *R*_1_ of the WB is active and constant current biased by *I_ref_*. The equivalent circuit is shown in Figure 2b, where the active element is named, in a general case, *R_s_* (*s* = 1 in this particular case).

### 2.1. MR-Shunt Definition and Its Electronic Interface

In Figure 2b, the action of an external magnetic field *H*, generated by the current *i* to be measured, according to the MR effect [25,26,27], results in a magnetic resistance change (Δ*R_s_*) proportional to the applied magnetic field within its linear range and, therefore, proportional to the current to be measured:

(1) $$\Delta R_{s}=R_{s}-R_{os}=S_{\Omega }\cdot i$$

with $S_{\Omega }$ (in Ω/A) being the resistive sensitivity of the MR-shunt and *R_os_* the sensor resistance at zero input current.

To convert the change of resistance into voltage, the MR-shunt *R_s_* in Figure 2b is biased at a constant current *I_ref_* and the voltage *v* measured:

(2) $$v=I_{ref}\cdot \left(R_{os}+\Delta R_{s}\right)=I_{ref}\cdot \left[R_{os}+S_{\Omega }\cdot i\right]=S\cdot i+V_{off}$$

with *S* being the sensor voltage sensitivity (in V/A), which depends on the sensor type and the supplied current *I_ref_*,

(3) $$S=I_{\mathrm{ref}}\cdot S_{\Omega }$$

and *V_off_* the voltage at zero input current,

(4) $$V_{off}=I_{ref}\cdot R_{os}.$$

**Figure 3:**
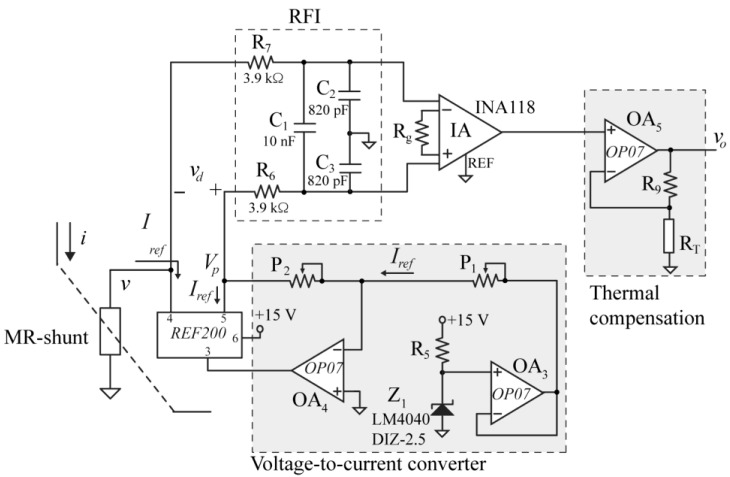
Electronic circuitry for MR-shunt temperature compensation.

The conditioning circuit used for the MR-shunt is shown in Figure 3. It is based on a voltage-to-current converter (*OA*_3_ and *OA*_4_) that provides the reference current *I_ref_* and a current mirror (REF200 from Texas Instruments, Dallas, TX, USA) that replicates this current in the MR-shunt. The current *I_ref_* is set by the voltage reference *Z*_1_ (2.5 V) and *P*_1_ that adjusts it to the desired value (e.g., 1 mA). The differential voltage *v_d_* in Figure 3, by considering *R_s_* of (1), is:

(5) $$v_{d}=I_{ref}\cdot \left(R_{os}+\Delta R_{s}-P_{2}\right).$$

For zero current *i* (i.e., $\Delta R_{s}=0$), *P*_2_ allows adjusting the voltage $V_{p}=-I_{ref}\cdot P_{2}\;$ in Figure 3 to the offset voltage (*V_off_*) of Equation (4), in such a way that *v_d_* is equal to zero. Therefore, after offset adjustment:

(6) $$v_{d}=I_{ref}\cdot \Delta R_{s}=S\cdot i.$$

Next, the differential voltage *v_d_* is Radio Frequency Interference (RFI)-filtered using the *R*_6_-*R*_7_-*C*_1_-*C*_2_-*C*_3_ network. This prevents any RFI content from contributing to an offset voltage at the output of the instrumentation amplifier that will not be eliminated by subsequent low-pass filtering [28]. In this design, there is a cutoff frequency for the differential mode of 2 kHz ($f_{c,dm}=\frac{1}{2\pi \left(2C_{1}+C_{3}\right)R_{6}}$) and for the common mode of 50 kHz ($f_{c,cm}=\frac{1}{2\pi R_{6}C_{3}}$). The resulting signal is then conditioned by an instrumentation amplifier (*IA*), with gain *G*, and a non-inverter amplifier (*OA*_5_) to obtain the output voltage *v_o_*.

### 2.2. MR-Shunt Compensation Method

The compensation method described below is based on imposing a zero-temperature coefficient to the variable to compensate, which gives a design condition for the compensation circuit. This technique has been successfully applied in force, pressure, or magnetic field sensor bridges, as well as in the design of precision voltage references [29,30,31].

If a thermal dependence of the sensitivity *S* is considered in the form:

(7) $$S=S_{o}\cdot \left[1+TCS\cdot \left(T-T_{o}\right)\right]$$

where *S_o_* and *TCS* are, respectively, the sensor sensitivity and its temperature coefficient at temperature *T_o_*, then Equation (6) can be written as:

(8) $$v_{d}=S_{o}\cdot \left[1+TCS\cdot \left(T-T_{o}\right)\right]\cdot i$$

so that the temperature coefficient of *v_d_* at *T = T_o_* is:

(9) $$TCv_{d}=TCS.$$

To compensate for the *v_d_* variation in temperature, a temperature-variable resistor *R_T_* is added in the gain of the non-inverting amplifier (Figure 3). In this way, its output voltage *v_o_* will be given by:

(10) $$v_{o}=G_{n,inv}\cdot G\cdot v_{d}$$

where:

(11) $$G_{n,inv}\left(T\right)=1+\frac{R_{9}}{R_{T}\left(T\right)}.$$

By applying natural logarithms in Equation (10) and then differentiating, it is possible to obtain the expression:

(12) $$\frac{dv_{o}}{v_{o}}=\frac{dG_{n,inv}}{G_{n,inv}}+\frac{dv_{d}}{v_{d}}$$

with *G* being constant with temperature.

If derivatives are now calculated with respect to temperature, to achieve a *TCv_o_ =* 0 at temperature *T_o_*, the following condition must be met:

(13) $$TCG_{n,inv}=-TCv_{d}=-TCS.$$

From Equation (11), it is possible to obtain, for *TCG_n_*_,*inv*_:

(14) TCGn,inv=dGn,invdTGn,inv=−R9R9+RT(To)·TCRT(T).

Thus, from Equation (13), a specific value for resistance *R*_9_ is obtained at a given compensation temperature:

(15) $$R_{9}=\frac{R_{T}\left(T_{o}\right)}{\frac{TCR_{T}\left(T\right)}{TCS}-1}.$$

To obtain a physically feasible value of *R*_9_, it will be necessary that *TCR_T_* > *TCS*.

## 3. Experimental Results

To validate the proposed electronic interface and the effectiveness of the compensation method, two MR-shunt elements were subjected to an *i* current sweep. The first one was integrated into a WB with the connections indicated in Figure 2a and the second element implemented autonomously.

### 3.1. Electronic Interface Validation

**Figure 4:**
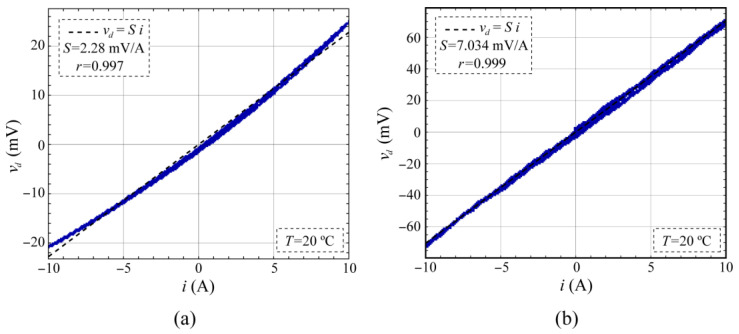
Voltage difference *v_d_* corresponding to a current sweep from −10 A to +10 A. (**a**) MR element embedded in a Wheatstone bridge (TMR46) and (**b**) stand-alone element (LCEL1), *T* = 20 °C.

The MR-shunt element was biased at a constant current value of *I_ref_* = 1 mA with a +2.5 V reference voltage (LM4040DIZ from Texas Instruments, Dallas, TX, USA) and adjusting potentiometer *P*_1_. By means of a transconductance amplifier (PCS-2B from Krohn-Hite Corporation, Brockton, MA, USA), a current sweep from −10 A to +10 A was generated in a PC-controlled manner and the MR-shunt voltage *v_d_* was acquired using a controlled multimeter (K2000 from Keithley, Solon, OH, USA). Potentiometer *P*_2_ was adjusted to achieve zero *v_d_* output at zero current *i*. Figure 4 shows the experimental responses of the *v_d_* voltage corresponding to the MR-shunt element embedded in the Wheatstone bridge (Figure 4a) and the single element (Figure 4b) at a temperature *T* = 20 °C.

In Figure 4, a high degree of linearity is obtained in both the MR-shunt element embedded in the Wheatstone bridge (TMR46) and the single element (LCEL1). However, the linearity is slightly higher in the LCEL1 element than in the TMR46, probably due to a residual influence of the other three MR elements present in the Wheatstone bridge configuration. In a potential application, the practical linear range of the embedded element should be reduced and the use of the isolated MR element would be even more advisable. Moreover, a higher sensitivity for the LCEL1 element was also observed than that for the TMR46 due to its particular manufacturing structures.

### 3.2. Sensitivity Temperature Coefficients without Compensation

**Figure 5:**
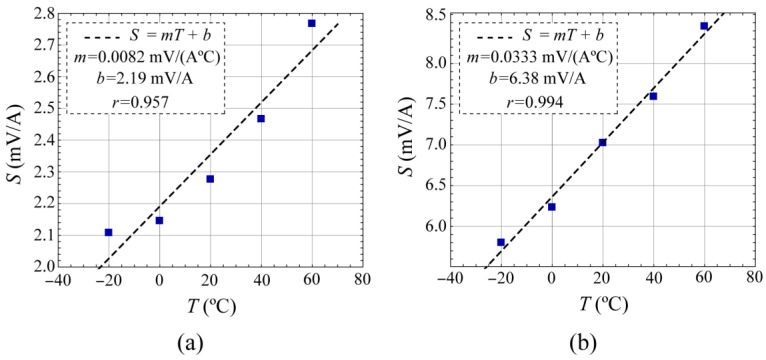
Temperature dependence of the current sensitivity *S* corresponding to: (**a**) MR element embedded in a Wheatstone bridge (TMR46) and (**b**) single element (LCEL1).

Similar to the methodology employed in the previous section, voltage *v_d_* was measured in response to the *i* current sweep at different temperatures by placing the MR element within a climatic chamber (CH600 VT from Angelantoni, Massa Martana, Italy). Thermal analysis was performed in the temperature range of −20 to 60 °C. The current sensitivity *S* for voltage *v_d_* at each temperature was subsequently analyzed through linear regression, similar to the way as shown in Figure 4. The change in *S* with temperature is shown in Figure 5.

Temperature dependence of the current sensitivity *S* shows a good linearity with a slope *m* of 0.0082 mV/A°C for the TMR46 and 0.0333 mV/A°C for the LCEL1, which is equivalent at *T_o_* = 20 °C to a *TCS* of 0.3482%/°C and 0.4743%/°C, respectively. Moreover, a higher temperature dependence and degree of linearity are shown for the single element LCEL1, according to its greater sensitivity and better linearity (see Figure 4b).

### 3.3. Sensitivity Temperature Coefficients with Compensation

Once thermal characterization of the two MR elements had been carried out, the aim was to minimize the thermal dependence of their electronic interface (voltage *v_o_* in Figure 3). The objective was to decrease the gain of the non-inverting stage as the temperature rises, thus compensating for the temperature-induced increase in sensitivity of the MR sensor. In this way, resistance *R_T_* must be a temperature sensor with positive TC, and the value of *R_9_* will be determined by Equation (15) obtained in the compensation method (Section 2.2).

For *R_T_*, four possible temperature sensors were considered and thermally characterized. Three of them were resistive temperature detectors (RTDs), two platinum-based (Pt100 and Pt1k), and one Ruthenium-based (Ru), while the fourth was a silicon-based temperature sensor (the KTY81-122 model [32,33] from NXP Semiconductors, Eindhoven, The Netherlands). Experimental measurements for each sensor provided the temperature coefficients outlined in Table 1.

According to Equation (15), suitable resistance values for *R*_9_ were obtained using the Pt1k and KTY81-122 sensors for the embedded element TMR46 and the KTY81-122 sensor for the single element LCEL1.

In a similar way to measurement of the sensing elements, the voltage *v_o_* measurements were carried out at the temperatures outlined in Section 3.2, performing the current sweep detailed in Section 3.1.

**Figure 6:**
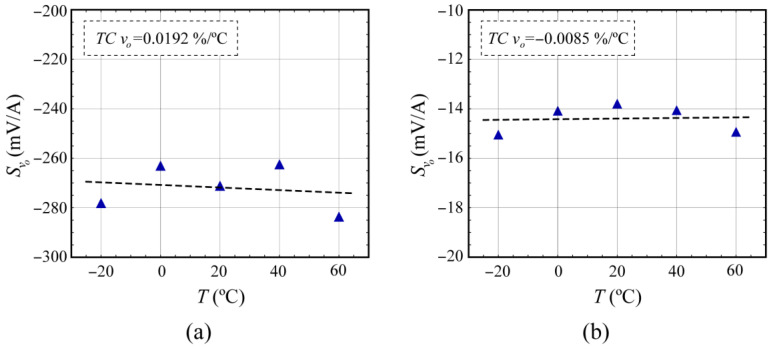
Temperature dependence of the current sensitivity *S_vo_* of the voltage *v_o_* at the output of the electronic interface corresponding to the MR element embedded in a Wheatstone bridge (TMR46) using the temperature sensors (**a**) RTD-Pt1k and (**b**) silicon-based KTY81-122.

**Figure 7:**
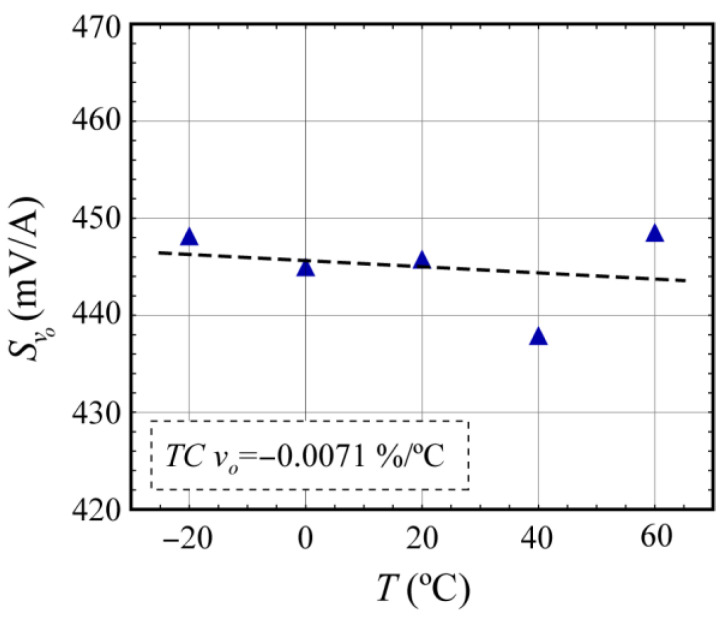
Temperature dependence of the current sensitivity *S_vo_* of the voltage *v_o_* at the output of the electronic interface corresponding to the stand-alone element (LCEL1) using the silicon-based temperature sensor KTY81-122.

Figure 6 and Figure 7 show the values of the current sensitivity *S_vo_* of the voltage *v_o_* at the output of the electronic interface at different temperatures for the TMR46 and the LCEL1 MR-shunts, respectively, after temperature compensation. The main results are summarized in Table 2, where *TC_wnc_* denotes the temperature coefficients obtained without compensation (Section 3.2) and *TC_wc_* with compensation (Table 2).

A figure of merit that reflects the effectiveness of the thermal compensation performed is the percentage in reduction *R* experienced in the temperature coefficient before and after compensation, defined as:

(16) $$R\left(\%\right)=\left(1-\left|\frac{TC_{wc}}{TC_{wnc}}\right|\right)\cdot 100$$

As summarized in Table 2, it was possible to reduce the initial temperature coefficients of the MR-shunt elements, achieving high reduction coefficients in all the cases tested, especially when the silicon-based temperature sensor was used.

## 4. Discussion

The temperature coefficient of MR-shunt elements can be hardware-compensated by the use of a suitable conditioning circuit (Figure 3) and a specific temperature sensor (compensator). The proposed compensation method is based on the variation of voltage gain with temperature and obtains a resistance whose realizability depends on the relationship between temperature coefficients of the compensator and the MR-shunt element (Equation (15)). Therefore, it is convenient to use compensators with high temperature coefficients, like those implemented from silicon, such as the one described in the work [32] (0.790%/°C) or the type of RTDs based on Pt (0.353%/°C). Regarding these two compensators, Figure 6 and Figure 7 show a more optimal thermal compensation when using the silicon-based compensator. Higher *TCR_T_* values result in a resistance *R_9_* that is less sensitive to changes in the temperature coefficient of the compensator, particularly away from negative values and the point of indetermination, thus achieving better compensation. RTDs based on Ni with TC values around 0.618%/°C [34,35] or linear thermistors [36] would also be suitable but have not been tested in this work.

In the case of the MR-element embedded in the Wheatstone bridge, the other three MR elements present in it affect the results. The magnetic behavior of an MR element is not the same when it is joined to the other three by means of a Wheatstone bridge connection than when it is alone. In Figure 2, as was previously explained, the operational amplifiers *OA*_1_ and *OA*_2_ establish a zero-voltage difference in the adjacent elements *R*_2_ and *R*_3_, blocking the current *I_ref_* through them, and thus preventing their polarization. However, in real operational amplifiers, an offset voltage exists that gives a non-zero voltage difference between their input terminals. Consequently, the voltage difference in *R*_2_ and *R*_3_ differs from zero in this offset voltage, thus allowing a residual polarization in these components. This residual polarization is probably the cause of less linearity of this configuration, as evidenced by the experimental data presented in Figure 4a and Figure 5a. In the present work, ultralow offset voltage operational amplifiers *OA*_1_ and *OA*_2_ were selected to minimize the residual polarization of *R*_2_ and *R*_3_ elements to a level less than 0.1 μA. Figure 4a and Figure 5a depict how residual polarization impacts the linear behavior of the embedded MR element and its temperature sensitivity. The use of a single MR element enhances the linearity of its behavior, as evidenced by the results presented in Figure 4b and Figure 5b.

Compensations of current sensors in Wheatstone bridge configuration based on spin-valve technology were presented in [37,38,39]. For these cases, a compensator based on an Ru RTD sensor was integrated into the same substrate as the sensor bridge. In that situation, the bridge supply current varied with temperature. In the design presented, it is the gain of a voltage amplifier that changes with temperature and the TC of an Ru-based compensator was not sufficient to satisfy Equation (15). It would be highly desirable to have MR-shunt elements and Ru-based compensators sharing the same substrate to enhance thermal coupling between them, thereby enabling better thermal compensation using methods as described in [37,38,39].

## 5. Conclusions

A magnetoresistance-based current sensor has been presented that, on a practical level, offers the simplicity of a shunt resistor, while avoiding drawbacks such as galvanic isolation and self-heating issues. In this way, the concept of a magnetoresistive shunt (MR-shunt) is presented. The design replaces the conventional Wheatstone bridge structure used in magnetoresistive sensors with just a single MR element. This not only simplifies the microelectronic processes but also reduces the layout complexity of connections. Furthermore, the proposed electronic system includes compensation for the sensitivity thermal drift commonly associated with these sensors.

For the analyzed sensors, linear response behavior was obtained in the measurement range for currents between −10 and 10 A. Furthermore, by adding an external resistive temperature sensor (Pt1k or KTY) and with the proposed circuitry, a strong reduction in thermal dependence of the sensitivity was achieved (*R* > 94%, Table 2).

## Figures and Tables

**Table 1:** Temperature coefficients in %/°C of the three temperature sensors used for compensation.

Temperature Sensor	*TC* (%/°C) (*T_o_* = 20 °C)
RTD-Pt100	0.342
RTD-Pt1k	0.353
Ru	0.188
KTY81-122	0.758

**Table 2:** Temperature coefficients in %/°C without and with compensation, and the percentage of reduction *R* (%).

MR-Shunt	*TC_wnc_* (%/°C)	*TC_wc_* (%/°C)		*R* (%)	
		Pt1k	KTY	Pt1k	KTY
TMR46	0.3482	0.0192	−0.0086	94.5	97.5
LCEL1	0.4743	-	−0.0071	-	98.5

## Data Availability

Data are contained within the article.
